# Supplementary figures and images for: A national household survey on HIV prevalence and clinical cascade among children aged ≤15 years in Kenya (2018)
Source: PLoS One. 2022 Nov 23;17(11):e0277613. doi: 10.1371/journal.pone.0277613 (PMC9683548; doi:10.1371/journal.pone.0277613)

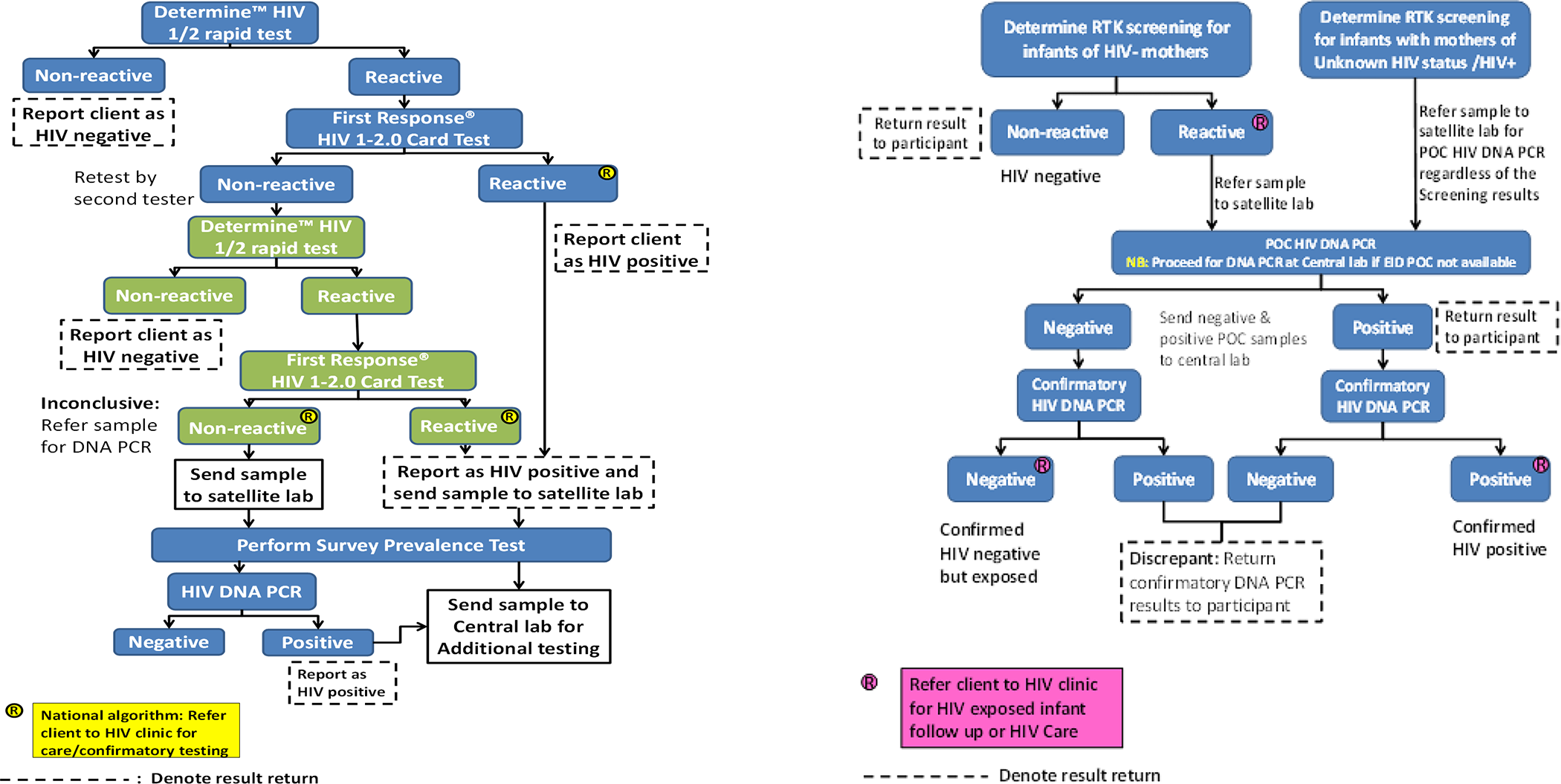

Supplement: S1 Fig — a. HIV testing algorithm for participants 18 months–14 years old, KENPHIA, 2018. b. HIV testing algorithm for participants <18months old, KENPHIA, 2018. (TIF) [file pone.0277613.s001.tif]

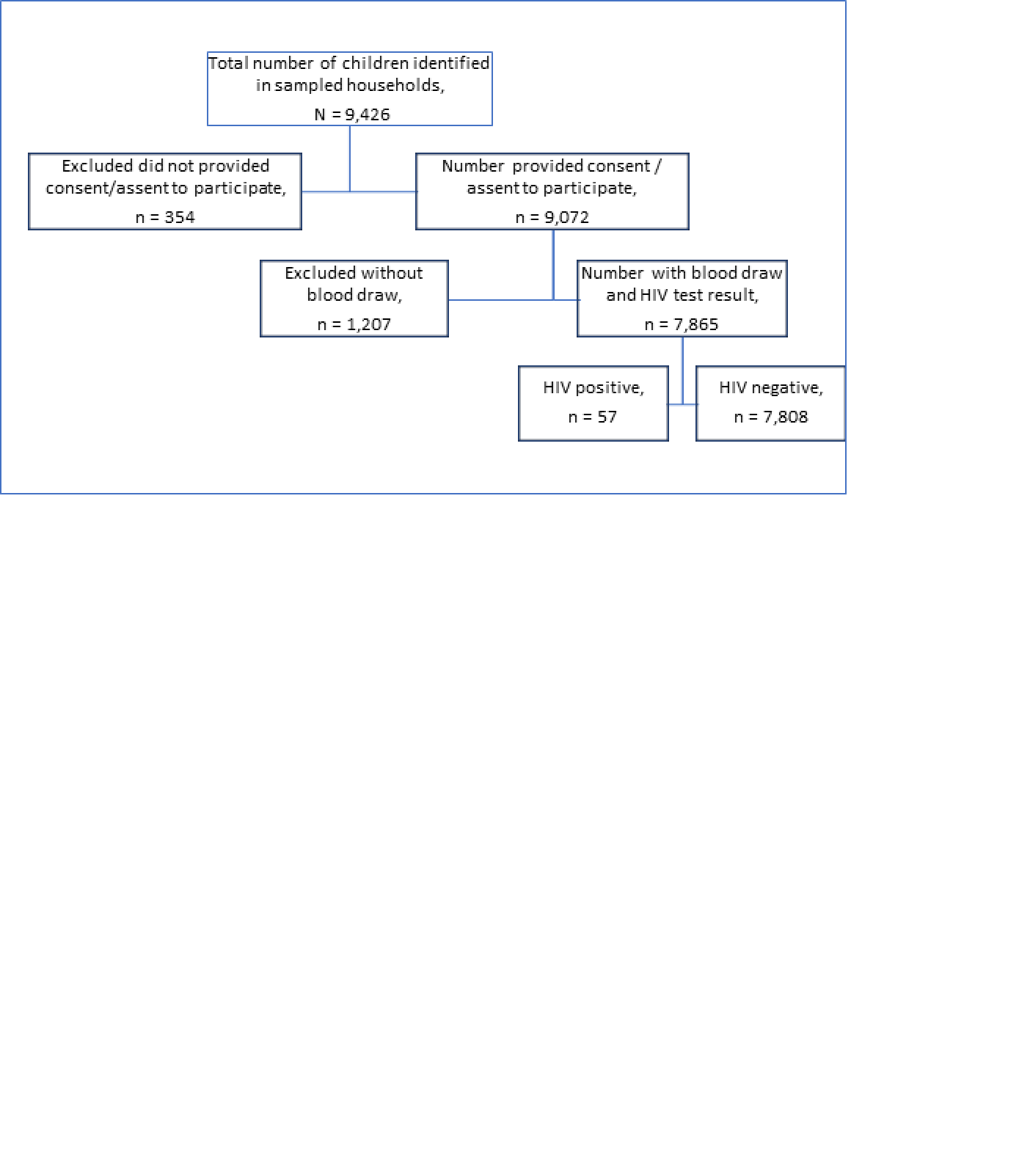

Supplement: S2 Fig — (TIF) [file pone.0277613.s002.tif]
